# Maternal serum retinol and β-carotene concentrations and neonatal bone mineralization: results from the Southampton Women’s Survey cohort^1^

**DOI:** 10.3945/ajcn.116.130146

**Published:** 2016-09-14

**Authors:** Mina N Händel, Rebecca J Moon, Philip Titcombe, Bo Abrahamsen, Berit L Heitmann, Philip C Calder, Elaine M Dennison, Sian M Robinson, Keith M Godfrey, Hazel M Inskip, Cyrus Cooper, Nicholas C Harvey

**Affiliations:** 2 National Institute of Public Health and; 3 Department of Clinical Research, Odense Patient Data Explorative Network, Odense University Hospital, University of Southern Denmark, Odense, Denmark;; 4 Research Unit for Dietary Studies, Parker Institute and the Institute of Preventive Medicine, Bispebjerg and Frederiksberg University Hospital, Copenhagen, Denmark;; 5 Medical Research Council Lifecourse Epidemiology Unit,; 6 National Institute for Health Research Southampton Biomedical Research Centre, University Hospital Southampton National Health Service Foundation Trust, and; 7 Human Development and Health Academic Unit, Faculty of Medicine, University of Southampton, Southampton, United Kingdom;; 8 Department of Medicine, Holbæk Hospital, Holbæk, Denmark;; 9 Section of General Practice, Department of Public Health, University of Copenhagen, Copenhagen Denmark; and; 10 National Institute for Health Research Musculoskeletal Biomedical Research Unit, University of Oxford, Oxford, United Kingdom

**Keywords:** bone development, epidemiology, pregnancy, vitamin A, retinol, β-carotene

## Abstract

**Background:** Studies in older adults and animals have suggested contrasting relations between bone health and different vitamin A compounds. To our knowledge, the associations between maternal vitamin A status and offspring bone development have not previously been elucidated.

**Objective:** We examined the associations between maternal serum retinol and β-carotene concentrations during late pregnancy and offspring bone mineralization assessed at birth with the use of dual-energy X-ray absorptiometry.

**Design:** In the Southampton Women’s Survey mother-offspring birth cohort, maternal health, lifestyle, and diet were assessed prepregnancy and at 11 and 34 wk of gestation. In late pregnancy, maternal serum retinol and β-carotene concentrations were measured. Offspring total body bone mineral density (BMD), bone mineral content (BMC), and bone area (BA) were measured within 2 wk after birth.

**Results:** In total, 520 and 446 mother-offspring pairs had measurements of maternal serum retinol and β-carotene, respectively. Higher maternal serum retinol in late pregnancy was associated with lower offspring total body BMC (β = −0.10 SD/SD; 95% CI: −0.19, −0.02; *P* = 0.020) and BA (β = −0.12 SD/SD; 95% CI: −0.20, −0.03; *P* = 0.009) but not BMD. Conversely, higher maternal serum β-carotene concentrations in late pregnancy were associated with greater total body BMC (β = 0.12 SD/SD; 95% CI: 0.02, 0.21; *P* = 0.016) and BA (β = 0.12 SD/SD; 95% CI: 0.03, 0.22; *P* = 0.010) but not BMD.

**Conclusions:** Maternal serum retinol and β-carotene concentrations had differing associations with offspring bone size and growth at birth: retinol was negatively associated with these measurements, whereas β-carotene was positively associated. These findings highlight the need for further investigation of the effects of maternal retinol and carotenoid status on offspring bone development.

## INTRODUCTION

Identifying novel strategies to improve bone health is important in addressing the high incidence of both pediatric fractures (1) and fragility fractures in adult life (2). One approach might be to target pregnant women to influence early fetal bone development. Indeed, in observational studies, offspring bone health is associated with maternal lifestyle (3) and dietary characteristics during pregnancy (4). Vitamin A comprises a group of fat-soluble essential nutrients that have roles in vision, immune function, growth, and cell division and differentiation (5). Different dietary sources yield different forms of vitamin A. Specifically, plant sources contain provitamin A carotenoids, of which the most common is β-carotene, and animal sources provide preformed vitamin A, such as retinoids and retinyl esters (5). There is evidence in adults that vitamin A intake is associated with measures of bone health, but notably, the different forms of dietary vitamin A have been demonstrated to have opposing associations with fracture risk (6). Thus, in a recent meta-analysis, high intakes of retinol among adults were associated with an increased risk of osteoporosis and fragility fractures (6). Similar findings have been observed in animal studies (7–9). In contrast, in the same meta-analysis, high β-carotene intake was suggested to be associated with lower fracture risk (6).

Few studies to our knowledge have related maternal vitamin A nutrition during pregnancy to offspring bone development. In the prospective Avon Longitudinal Study of Parents and Children cohort study, maternal dietary intakes of retinol and carotene, measured by a food-frequency questionnaire (FFQ)^11^ at 32 wk of gestation, were not associated with offspring whole-body bone mineral content (BMC), bone mineral density (BMD), or size-corrected BMC (scBMC) assessed by dual-energy X-ray absorptiometry (DXA) in children aged 9 y (10). However, FFQs can be prone to measurement error and thus may have resulted in misclassification. Indeed, the correlation between FFQ measurements of retinol and carotene intake in pregnancy and biochemical assessments of status has been found to be weak (11), although this may in part also reflect the insensitivity of plasma concentrations to intake (12). The objective of this study was therefore to explore the associations between maternal serum retinol and β-carotene concentrations during late pregnancy and offspring bone mineralization at birth in the SWS (Southampton Women’s Survey) cohort. We hypothesized that maternal serum retinol and β-carotene concentrations would show opposing associations with neonatal bone outcomes.

## METHODS

### SWS

**FIGURE 1:**
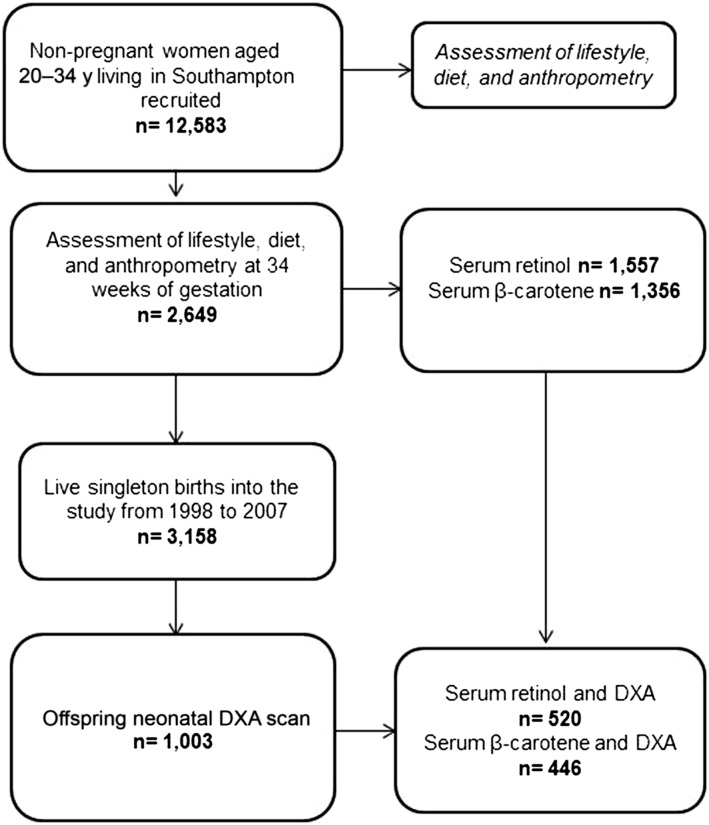
Participant flowchart. DXA, dual-energy X-ray absorptiometry.

SWS is a prospective mother-offspring birth cohort study (13). Nonpregnant women aged 20–34 y and residing in Southampton, United Kingdom (*n* = 12,583), were initially recruited into the study. Thereafter, there were 3158 live-born singleton births to women in the study between 1998 and 2007. A flowchart of the study population, for whom there were maternal serum retinol and β-carotene concentration data and neonatal bone measures, is presented in **Figure 1**.

SWS was conducted according to Declaration of Helsinki guidelines, and the Southampton and South West Hampshire Research Ethics Committee approved all procedures. Written informed consent was obtained from all participating women.

### Maternal assessments

At study entry (prepregnancy), early pregnancy (∼11 wk of gestation), and late pregnancy (34 wk of gestation), research nurses administered questionnaires on maternal demographics, lifestyle, and health. The highest level of educational attainment was recorded. Diet was assessed with the use of a validated 100-item FFQ, and anthropometric measurements were obtained (13, 14). With the use of principal component analysis, the FFQ was used to calculate a measure of dietary quality termed “prudent diet score.” A higher prudent diet score indicates a healthier dietary quality, including high intakes of fruits, vegetables, and whole-meal bread, rice and pasta, and low intakes of processed foods (15). In a previous study of 585 pregnant women, prudent diet scores calculated with the use of prospective food diaries from this FFQ were highly correlated (*r* = 0.67) (14).

### Biochemical analyses

A maternal blood sample was obtained during the late-pregnancy assessment. The serum was stored at −80°C until analysis to determine maternal serum concentrations of retinol and β-carotene. The sample measurements were carried out in the laboratory of the Institute of Human Nutrition, University of Southampton, based on the existing birth cohort at the end of 2003. HPLC with multiwavelength spectrophotometric detection was used to analyze retinol and β-carotene. The HPLC equipment consisted of a Beckman Coulter System Gold 125 Solvent System and 508 Autosampler connected to a Supelcosil C18 reverse-phase column (25 × 0.46 cm; 5-μm particle size) (Sigma-Aldrich). Retinol and β-carotene were measured by absorbance at 325 and 460 nm, respectively. Standards of retinol and β-carotene were prepared daily from stock solutions stored at −80°C, and their concentrations were determined by spectrophotometry. Acceptable accuracy and precision were confirmed by the use of frozen aliquots of pooled serum. The CVs were between 5% and 10%. Maternal vitamin D status was determined from serum 25-hydroxyvitamin D concentrations. These were analyzed by radioimmunoassay (Diasorin). This assay measures 25-hydroxyvitamins D_2_ and D_3_ and meets the requirements of the Vitamin D External Quality Assurance Scheme. Intra- and interassay CVs were <10%.

### Offspring assessments

Mothers registered with selected general practitioner practices were invited to participate in the SWS bone substudy. DXA scans of neonatal total body BMC, bone area (BA), and BMD were obtained within 2 wk after birth with the use of a Lunar DPX-L instrument with specific pediatric software (pediatric small-scan mode, version 4.7c; GE Corporation). Daily quality assessment and weekly calibration against a water phantom were performed. Measurements were standardized with regard to the child being pacified, fed if necessary, wearing no clothing and placed on a waterproof sheet in a standard position on the scanner. The child was kept in place with the use of rice bags placed over the bottom end of the towel. The short- and long-term CVs of the DXA instruments were 0.8% and 1.4%, respectively.

### Statistical methods

Scatter plots were initially used to visualize the relation between retinol or β-carotene and offspring total body BMC, BA, and BMD. Associations were tested for nonlinearity, and linear regression was used with retinol, β-carotene, and β-carotene:retinol ratio separately as the exposures and offspring bone and body composition as the outcomes. To avoid size-related artifacts, scBMC was calculated as an indicator of volumetric BMD by adjusting BMC for BA, weight, and crown-heel length (16). Two participants were missing an scBMC value because of a missing value for crown-heel length. All outcome and exposure variables were standardized to obtain normally distributed variables with a mean of 0 and SD of 1. Thus, associations are presented as standardized β-coefficients. Serum β-carotene values and β-carotene:retinol ratio were log_e_-transformed to normalize the distribution before standardization could be applied. Diagnostic plots were used to check for heteroscedasticity. Unpaired *t* tests were used to check for sex differences in the exposure and outcome variables of the offspring. The model was adjusted a priori for offspring sex, gestational age, age at DXA scan, maternal smoking during pregnancy, late-pregnancy walking speed, late-pregnancy triceps skinfold thickness, parity, and maternal educational status because these factors have previously been associated with neonatal bone mineralization (3). In a further analysis, we included maternal prudent diet score to investigate whether associations might be mediated via overall maternal dietary pattern. Statistical analysis was carried out with the use of Stata versions 13.1 and 14.0. (StataCorp LP). *P* < 0.05 was considered to be statistically significant.

## RESULTS

### Characteristics of the cohort

There were 520 and 446 mother-offspring pairs with maternal serum retinol and β-carotene measurements, respectively. The characteristics of the mothers who had serum concentrations of either retinol or β-carotene and an offspring neonatal DXA scan are presented in **Table 1** together with the characteristics of the remaining women in the cohort. Compared with mothers within the SWS cohort who had a live singleton birth but were not included in our analysis, mothers in our analysis were of similar age and had similar prepregnancy BMIs (in kg/m^2^), triceps skinfold thicknesses, and parity, but in our analysis fewer women smoked during pregnancy and they had attained a higher educational status. In total, <2% of the women studied took retinol-containing supplements in late pregnancy (median intake: 533 μg/d; IQR: 267, 723 μg/d); 7% took β-carotene-containing supplements (median intake: 3903 μg/d; IQR: 1666, 4199 μg/d). The use of vitamin A supplements was comparable for women included in this analysis and other women in the SWS cohort (Table 1). The characteristics of the offspring are presented in **Table 2**. Males had a lower gestational age at birth than females but a greater BMD (Table 2).

**TABLE 1:** Characteristics of SWS mothers^1^

	Mother-child pairs studied		Remaining mother-child pairs	
	Values	*n*	Values	*n*	*P* values
Age at delivery, y	30.4 ± 3.7^2^	523	30.7 ± 3.9	2633	0.06
Prepregnancy BMI, kg/m^2^	24.3 (22.0, 28.1)^3^	518	24.1 (21.8, 27.2)	2610	0.10
Late-pregnancy triceps skinfold thickness, mm^3^	20.8 (17.0, 25.7)	520	20.8 (16.5, 25.3)	2058	0.19
Late-pregnancy serum retinol, μmol/L	1.33 ± 0.35	520	1.31 ± 0.36	1032	0.41
Late-pregnancy serum β-carotene, μmol/L	0.29 (0.20, 0.45)	446	0.32 (0.22, 0.47)	905	0.05
Smoked during pregnancy, *n* (%)	69 (13.2)	523	416 (16.9)	2465	0.04
Nulliparous, *n* (%)	254 (48.6)	523	1357 (51.6)	2630	0.21
Took supplementary retinol in late pregnancy, *n* (%)	9 (1.7)	520	51 (2.4)	2122	0.36
Took supplementary β-carotene in late pregnancy, *n* (%)	34 (6.5)	520	194 (9.1)	2122	0.06
Educational status,^4^ *n* (%)		521		2626	0.02
None	9 (1.7)		90 (3.4)		
≤GCSE grade D	41 (7.9)		253 (9.6)		
≥GCSE grade C	143 (27.4)		767 (29.2)		
Advanced levels or equivalent	153 (29.4)		800 (30.5)		
HND	45 (8.6)		153 (5.8)		
Degree	130 (25.0)		563 (21.4)		

1 *P* values were used to compare maternal characteristics according to *t* tests for normally distributed continuous variables, Mann-Whitney rank-sum tests for nonnormally distributed continuous variables, and chi-square tests for categorical variables. GCSE, general certificate of secondary education; HND, higher national diploma; SWS, Southampton Women’s Survey.

2 Mean ± SD (all such values).

3 Median; IQR in parentheses (all such values).

4 GCSEs taken at the age of 16 y, advanced levels at the age of 18 y, and HNDs and degrees thereafter.

**TABLE 2:** Clinical characteristics of the offspring at birth^1^

	Females (*n* = 241)	Males (*n* = 282)	*P* values
Gestational age, wk	40.4 (39.3, 41.2)^2^	40.0 (39.1, 41.0)	0.002
Birth weight, kg	3.49 ± 0.52^3^	3.57 ± 0.48	0.064
Total body BMC, g	61.91 ± 16.25	64.47 ± 15.52	0.066
Total body BA, cm^2^	116.8 ± 27.1	120.5 ± 25.3	0.101
Total body BMD, g/cm^2^	0.527 ± 0.028	0.532 ± 0.026	0.040
Total body scBMC, kg	0.062 ± 0.003	0.062 ± 0.003	0.070

1 *P* values were used for comparisons according to *t* tests for birth weight and all bone measures and Mann-Whitney rank sum tests for gestational age. BA, bone area; BMC, bone mineral content; BMD, bone mineral density; scBMC, size-adjusted bone mineral content.

2 Median; IQR in parentheses (all such values).

3 Mean ± SD (all such values).

### Serum vitamin A (retinol and β-carotene) and neonatal bone indexes

**FIGURE 2:**
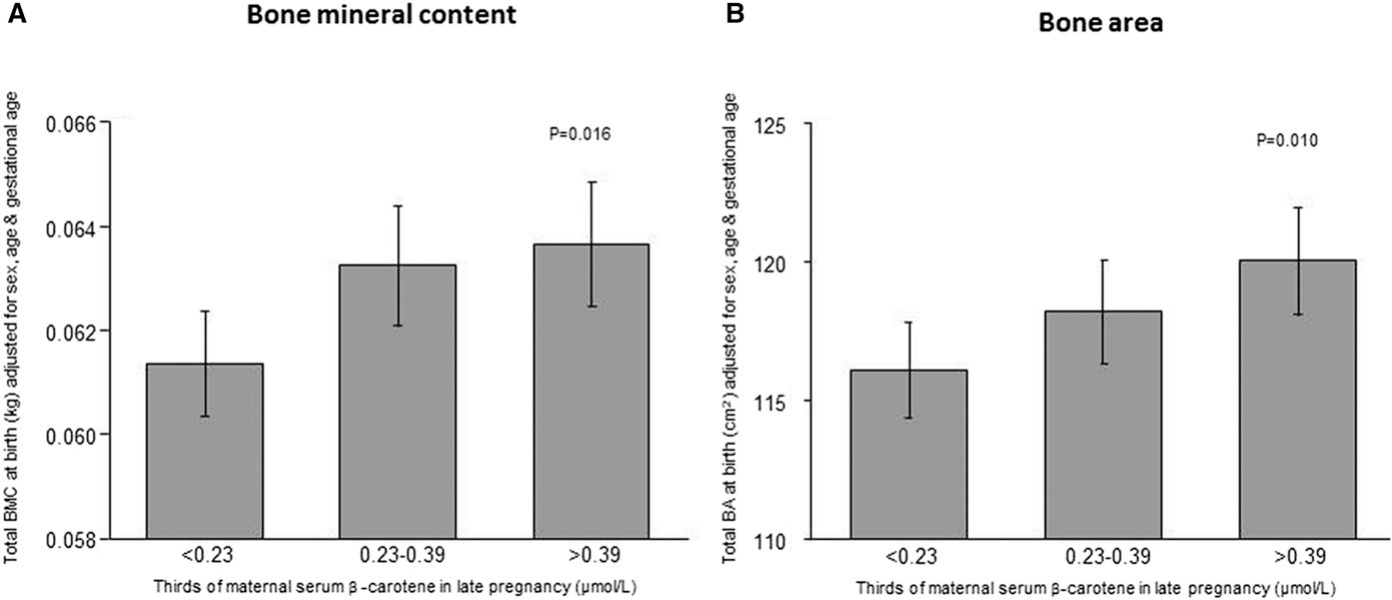
Offspring whole-body BMC (A) and BA (B) at birth according to thirds of maternal serum β-carotene concentrations in late pregnancy. Data are means ± SEMs. *P* values are for continuous relations assessed with the use of linear regression (*n* = 443 in both analyses). Results were adjusted for sex, age, and gestational age at birth. BA, bone area; BMC, bone mineral content.

After adjusting for maternal factors, gestational age, sex, and age at DXA scan, maternal serum retinol in late pregnancy was significantly negatively associated with offspring total body BMC, BA, and birth weight but not BMD or scBMC (**Table 3**). In contrast, maternal serum β-carotene concentration was positively associated with offspring total body BMC (*P* = 0.016) and BA (*P* = 0.010) at birth (**Figure 2**, Table 3), whereas there was no statistically significant association with offspring BMD, scBMC, or birth weight (Table 3). The maternal serum β-carotene:retinol ratio was positively associated with BA, BMC, and birth weight but not BMD and scBMC (**Table 3**). Including birth weight as a covariate markedly attenuated associations between measures of maternal vitamin A and offspring BA and BMC, and there was no evidence of any nonlinearity in the associations between vitamin A measures and offspring birth weight or bone indexes.

**TABLE 3:** Associations between serum retinol, β-carotene concentration, and β-carotene:retinol ratio in late pregnancy and bone measures at birth^1^

Bone measures at birth	Retinol	β-carotene	β-carotene:retinol ratio
Adjusted for sex, age, and gestational age			
Total body BMC	−0.10 (−0.19, −0.01)*	0.06 (−0.03, 0.15)	0.11 (0.02, 0.20)*
Total body BA	−0.12 (−0.20, −0.03)**	0.07 (−0.02, 0.16)	0.12 (0.03, 0.22)**
Total body BMD	0.01 (−0.08, 0.09)	0.02 (−0.07, 0.10)	0.03 (−0.05, 0.11)
Total body scBMC	0.03 (−0.06, 0.11)	−0.02 (−0.11, 0.07)	−0.03 (−0.12, 0.06)
Birth weight	−0.12 (−0.19, −0.05)***	0.04 (−0.03, 0.11)	0.10 (0.02, 0.17)**
Fully adjusted^2^			
Total body BMC	−0.10 (−0.19, −0.02)*	0.12 (0.02, 0.21)*	0.15 (0.06, 0.25)**
Total body BA	−0.12 (−0.20, −0.03)**	0.12 (0.03, 0.22)*	0.17 (0.07, 0.26)***
Total body BMD	−0.01 (−0.09, 0.08)	0.03 (−0.06, 0.12)	0.04 (−0.04, 0.13)
Total body scBMC	0.02 (−0.07, 0.11)	−0.02 (−0.11, 0.07)	−0.02 (−0.12, 0.07)
Birth weight	−0.11 (−0.18, −0.05)***	0.11 (0.02, 0.21)*	0.13 (0.05, 0.20)***

1 All values are standardized β-coefficients (SD/SD); 95% CIs in parentheses. BA, bone area; BMC, bone mineral content; BMD, bone mineral density; DXA, dual-energy X-ray absorptiometry; scBMC: size-adjusted bone mineral content.

2 Linear regression was used to adjust for sex, gestational age, age at DXA scan, maternal smoking during pregnancy, late-pregnancy walking speed, late-pregnancy triceps skinfold, parity, and maternal educational status: *P < 0.05, **P < 0.01, and ***P < 0.001.

### Maternal dietary pattern

There was a positive correlation between maternal serum β-carotene and late-pregnancy prudent diet score (*r*_s_ = 0.29; *P* < 0.0001) and a weaker correlation between maternal serum retinol and late-pregnancy prudent diet score (*r*_p_ = 0.14; *P* = 0.002). Including maternal prudent diet score in the linear regression model did not materially change the associations between β-carotene and offspring BMC and BA. In the final analyses, maternal vitamin D status in late pregnancy was included in the models. Adjusting for vitamin D status made little difference; the pattern of associations observed between serum retinol and β-carotene concentrations and neonatal bone indexes remained the same (data not shown).

## DISCUSSION

In this prospective mother-offspring cohort study, we observed differential associations between late-pregnancy serum retinol (negative) and β-carotene (positive) and offspring neonatal total BMC, BA at birth, and birth weight. We did not observe the same associations with BMD and scBMC. The relations were independent of maternal anthropometric, lifestyle, and socioeconomic factors and a measure of dietary quality.

### Comparison with previous literature

The observed associations with BA, BMC, and birth weight would suggest that maternal serum retinol and β-carotene might influence body size rather than having an independent effect on BMD. Previous intervention studies have not consistently demonstrated that prenatal vitamin A supplementation can increase birth size. However, these studies have typically been performed in developing countries with a high prevalence of vitamin A deficiency, and many have used an interventional product containing both retinol and β-carotene (17). Considering the opposing associations of retinol and β-carotene with birth weight observed in this study, it is possible that retinol and β-carotene used in combination had no overall effect.

There are few studies to our knowledge that have related maternal vitamin A status to offspring bone development. In the Avon Longitudinal Study of Parents and Children mother-offspring birth cohort study, maternal dietary retinol and β-carotene intake assessed by an FFQ at 32 wk of gestation were not significantly associated with offspring whole-body less head or lumbar spine BMC, BMD, or scBMC in children aged 9 y (10). However, the inaccuracies of FFQs in assessing intake should be considered. The relations between vitamin A nutrition, assessed by an FFQ or serum measurements, and fracture risk or BMD in adults are inconsistent (18), but in a recent meta-analysis Wu et al. (6) showed that higher intakes of retinol were associated with an increased risk of hip fracture, whereas higher β-carotene intake was suggested to be associated with lower hip fracture risk. Studies that have used serum measurements of retinol have suggested a U-shaped association with hip fracture risk (6), although there was no evidence of a nonlinear association between maternal serum retinol or β-carotene concentrations and offspring neonatal bone mineralization in this study.

Animal studies have shown that both high and low intakes of vitamin A can be detrimental to the bone. Rats with dietary-induced hypervitaminosis A caused by increased retinyl ester intakes have a smaller humeral cross-sectional area and cortical thinning than in controls but similar cortical and trabecular volumetric BMD (9). This is consistent with the negative associations between maternal serum retinol and offspring BA and BMC but not BMD observed in this study.

### Potential mechanisms

Studies in rats have shown that treatment with retinoids increases osteoclast number and markers of bone turnover (18, 19) but decreases osteoblastogenesis (8). Retinol has similarly been shown to increase resorption in fetal rat bones in culture (20), suggesting that the period of in utero development is also vulnerable to any potential bone effects of hypervitaminosis A. The mechanisms underlying the positive association between maternal β-carotene and offspring bone mineralization are less clear. However, the conversion of β-carotene to retinol is subject to negative feedback regulation; thus, the serum β-carotene:retinol concentrations, as opposed to the absolute concentrations of either, may be relevant (18). It is possible that a high serum β-carotene concentration reflects a dietary pattern rich in fruits and vegetables, which, in pregnancy, has previously been shown to be positively associated with offspring BMD. In the Pune Maternal Nutrition study in India, maternal fruit and green leafy vegetable intake during pregnancy was positively associated with offspring total body BMD in children aged 6 y (21), and in the Princess Anne Hospital Study in the United Kingdom, maternal prudent diet score was positively associated with offspring whole-body BMC in children aged 9 y (4). An elevated serum retinol concentration may represent a more Westernized diet high in animal foods, such as meat and meat products. In the Danish National Birth Cohort, offspring of mothers who consumed the most Westernized diets during pregnancy had the highest risk of forearm fractures in childhood (22). However, in this study, the observed associations between maternal β-carotene and offspring BMC and BA remained statistically significant after adjusting for maternal prudent diet score in pregnancy, suggesting that our findings are not explained by a higher serum β-carotene concentration, which reflects an overall healthier diet.

### Strengths and limitations

We studied a large prospective cohort and biochemically assessed serum retinol and β-carotene concentrations and DXA measures of offspring bone indexes. However, there are limitations that should be considered in the interpretation of our findings. First, retinol and β-carotene concentrations were measured in maternal blood, which correlates only weakly with umbilical cord blood measurements (23, 24). However, delivery is a period of high oxidative stress for infants, which could deplete antioxidant stores such as β-carotene; therefore, measurements in umbilical cord blood serum might not reflect fetal serum concentrations in utero. Although assessing liver reserves is the “gold-standard” measure of retinol status, it is not feasible in most human studies. Serum retinol concentration is commonly used instead, but it has limitations as an indicator of status (25). Nonetheless, our findings do suggest the need for further replicating other mother-offspring cohorts and potentially intervention studies with carotenoids in pregnancy. Second, women included in this analysis were less likely to have smoked during pregnancy and attained a higher level of education than in the SWS cohort as a whole. However, the mother-offspring pairs included in this analysis represent a diverse range of maternal ages and socioeconomic backgrounds. Furthermore, given that the analysis was within the cohort, there is no reason to expect that this would have erroneously led to the observed associations. Third, although DXA is well validated in adults, there are some problems in children because of their smaller size and tendency to move. Bone and body composition assessment by DXA of miniature piglets has been previously validated (26); we used specific pediatric software, and movement artifact was minimal. The few scans with excess movement artifact were excluded from the analysis. Finally, although it is a strength that we considered a range of potential confounding factors in our statistical models to describe the associations between maternal serum retinol and β-carotene concentrations and bone mineralization, these are observational data, and the possibility of residual confounding cannot be excluded.

### Conclusions

We have demonstrated that late-pregnancy serum retinol concentration is negatively associated with offspring bone size and mineralization at birth, whereas β-carotene is positively associated with these measurements. These findings may lend further support to dietary recommendations to limit retinol intake during pregnancy and highlight the potential for the further investigation of possible beneficial effects of carotenoids on offspring bone development.

## Footnotes

11 Abbreviations used: BA, bone area; BMC, bone mineral content; BMD, bone mineral density; DXA, dual-energy X-ray absorptiometry; FFQ, food-frequency questionnaire; scBMC, size-corrected bone mineral content; SWS, Southampton Women’s Survey.
